# Beta-Catenin and Epithelial Tumors: A Study Based on 374 Oropharyngeal Cancers

**DOI:** 10.1155/2014/948264

**Published:** 2014-01-08

**Authors:** Angela Santoro, Giuseppe Pannone, Silvana Papagerakis, H. Stan McGuff, Barbara Cafarelli, Silvia Lepore, Salvatore De Maria, Corrado Rubini, Marilena Mattoni, Stefania Staibano, Ernesto Mezza, Gaetano De Rosa, Gabriella Aquino, Simona Losito, Carla Loreto, Salvatore Crimi, Pantaleo Bufo, Lorenzo Lo Muzio

**Affiliations:** ^1^Department of Laboratory Medicine, Institute of Pathological Anatomy, Foundation for Research and Therapy “Giovanni Paolo II”, UCSC, 86100 Campobasso, Italy; ^2^Department of Clinical and Experimental Medicine, Section of Pathological Anatomy, University of Foggia, 71121 Foggia, Italy; ^3^Department of Otolaryngology, Head and Neck Surgery and Oncology, Medical School, University of Michigan, Ann Arbor, MI 48109, USA; ^4^Department of Pathology, Medical School, University of Texas Health Science Center at San Antonio, San Antonio, TX 78229, USA; ^5^Department of Economic Sciences, Mathematics and Statistics, University of Foggia, 71122 Foggia, Italy; ^6^Laboratory of Preclinical and Translational Research, IRCCS-CROB, Oncological Reference Centre of Basilicata, 85028 Rionero in Vulture, Italy; ^7^Institute of Biochemistry, SUN, 80100 Napoli, Italy; ^8^Section of Pathological Anatomy, Polytechnic University of Marche, 60100 Ancona, Italy; ^9^Section of Pathological Anatomy, Department of Advanced Biomedical Sciences, University of Napoli “Federico II”, 80100 Napoli, Italy; ^10^Section of Pathological Anatomy, National Cancer Institute “G. Pascale Foundation”, 80100 Napoli, Italy; ^11^Section of Anatomy and Histology, Department of Bio-Medical Sciences, University of Catania, 95100 Catania, Italy; ^12^Section of Maxillo-Facial Surgery, Department of Experimental Science in Medicine and Dentistry, University of Messina, 98100 Messina, Italy; ^13^IRCCS-CROB, Oncological Reference Centre of Basilicata, 85028 Rionero in Vulture, Italy; ^14^Section of Oral Pathology, Department of Clinical and Experimental Medicine, University of Foggia, 71122 Foggia, Italy

## Abstract

*Introduction*. Although altered regulation of the Wnt pathway via beta-catenin is a frequent event in several human cancers, its potential implications in oral/oropharyngeal squamous cell carcinomas (OSCC/OPSCC) are largely unexplored. Work purpose was to define association between beta-catenin expression and clinical-pathological parameters in 374 OSCCs/OP-SCCs by immunohistochemistry (IHC). *Materials and Methods*. Association between IHC detected patterns of protein expression and clinical-pathological parameters was assessed by statistical analysis and survival rates by Kaplan-Meier curves. Beta-catenin expression was also investigated in OSCC cell lines by Real-Time PCR. An additional analysis of the DNA content was performed on 22 representative OSCCs/OPSCCs by DNA-image-cytometric analysis. *Results and Discussion*. All carcinomas exhibited significant alterations of beta-catenin expression (*P* < 0.05). Beta-catenin protein was mainly detected in the cytoplasm of cancerous cells and only focal nuclear positivity was observed. Higher cytoplasmic expression correlated significantly with poor histological differentiation, advanced stage, and worst patient outcome (*P* < 0.05). By Real-Time PCR significant increase of beta-catenin mRNA was detected in OSCC cell lines and in 45% of surgical specimens. DNA ploidy study demonstrated high levels of aneuploidy in beta-catenin overexpressing carcinomas. *Conclusions*. This is the largest study reporting significant association between beta-catenin expression and clinical-pathological factors in patients with OSCCs/OPSCCs.

## 1. Introduction 

OSCCs and OPSCCs represent one of the major health issues, with over 200,000 new cases reported worldwide annually. Despite significant improvements in the early screening and diagnosis of these cancers, the 5-year-disease-free survival of those patients remains unacceptably poor. For many years, the main prognostic factors of SCC have been the conventional grading, staging, and site of tumor [1–3]. Nowadays, the goal of the scientific research is to find new biological markers able to define the “tumor biological fingerprint” and to identify the molecular key players that are involved in oropharyngeal carcinogenesis [4, 5]. Beta-catenin is a multifunctional protein expressed on the surface of epithelial cells, acting as a structural component of the E-cadherin-related cell adhesion system [6]. In this way beta-catenin has a crucial role in creation and maintenance of epithelial stability by regulating cell growth and adhesion between cells. Recent evidence suggests that beta-catenin also plays other important functional roles, including the control of cell polarization, differentiation, “stemness,” and cell motility [7, 8]. As a cause of genomic instability through aneuploidy, beta-catenin plays an important role in tumour development and aggressiveness [7–10]. Constitutive activation of the Wnt signaling pathway is a major etiological factor for many cancers [11–14]. Beta-catenin is the key mediator of the Wnt pathway. When the Wnt signaling is in its resting state, beta-catenin is phosphorylated by glycogen synthase kinase 3*β* (GSK3-*β*) within a protein complex that also includes casein kinase 1, adenomatous polyposis coli (APC), and Axin. Phosphorylated beta-catenin is immediately degraded via the ubiquitin proteasome pathway. WNT binding to Frizzled (Fz) results in the activation of Dishevelled (Dsh), which inhibits the activity of GSK3-*β*, resulting in dephosphorylation and stabilization of beta-catenin, enabling it to accumulate within the nucleus, where it interacts with members of the T-cell factor/lymphocyte enhancer factor (TCF/LEF) family and activate the expression of critical target genes governing cell fate, proliferation, and other processes in several types of cancer [15–20]. Moreover, although the role of the epithelial-to-mesenchymal transition (EMT) phenomenon in development and/or tumor progression and metastasis is still controversial, it is well known that beta-catenin and the cadherin switching can have a profound effect on cell phenotype and behavior [21, 22]. Previous studies have shown that CTNNB1, the gene of beta-catenin, together with APC and Axin are frequently mutated in different types of human cancers [7]. Although mutations in APC or CTNNB1 are rarely found in OSCC cell lines and in OSCC, catenin delocalization has been reported in premalignant and malignant lesions of the oral cavity and in OSCC [23, 24]. Other studies have demonstrated that aberrant expression of beta-catenin is inversely correlated with differentiation and is significantly associated with invasion and poor prognosis [25, 26]. In addition, we have recently reported that the cytoplasmic/nuclear accumulation of beta-catenin could be considered the final step of constitutive activation of WNT signal by epigenetic methylation of WNT inhibitors [27].

The aim of the present study, that retrospectively investigates a large series of oral and oropharyngeal carcinomas, occurred in Italy and North America, is to evaluate the expression profile of beta-catenin in OSCC/OPSCC and to assess its association with clinical and histopathological parameters.

## 2. Materials and Methods 

### 2.1. Study Population

Upon approval by the Ethical Committees of all the participants institutions, we identified 374 OSCCs/OPSCCs, selected from the Anatomic Pathology informatics archives of three different hospitals: “Ospedale Regionale Torrette—Ancona (1990–2007),” “Istituto Nazionale per lo studio e la cura dei Tumori G. Pascale”—Napoli (1996–2009), and the Department of Pathology, Medical School, University of Texas Health Science Center at San Antonio, Texas (1995–2007). Patients from these distinct geographical areas were previously untreated and received surgical and/or radio/chemotherapeutic treatment only with curative intention. Samples of human oropharyngeal cancers were obtained with the signed informed consent of all patients. Demographical and clinical-pathological characteristics of the study population were summarized in Table 1. The mean followup time of the studied cases was 39.41 months. Survival rate was calculated from the time of diagnosis to the date of the latest clinical follow-up or to patient's death due to disease or to other causes. An adequate series of normal oral and oropharyngeal mucosa specimens was used as controls for the comparative analyses. Finally, in 22 representative OSCCs one fragment of the tissue was immediately fresh frozen in liquid nitrogen and stored at 80°C for quantitative analysis by Real Time RT-PCR. The residual tissue was fixed in formalin solution for the routine histopathological examination and DNA ploidy analysis.

### 2.2. Immunohistochemistry

374 OSCCs/OPSCCs were quantitatively and qualitatively analyzed by immunohistochemistry for beta-catenin expression. Immunohistochemical analysis was performed by using Ventana Benchmark autostainer and/or manual standard linked streptavidin-biotin horseradish peroxidase technique (LSAB-HRP), according to the best protocol for the antibody tested in our laboratory and as previously described [28–30]. We used primary monoclonal rabbit anti-beta-catenin (clone 6B3; Cell Signaling Technology, Danvers, MA) diluted 1 : 150 and incubated overnight. Negative control slides without primary antibody were included for each staining. The results of the immunohistochemical staining were evaluated separately by two observers. Immunostained cells were counted in at least 10 high power fields (HPF) analyzed with an optical microscope (OLYMPUS BX41, at x40). The topographical staining pattern was evaluated and recorded as nuclear (N), membranous (M), cytoplasmic (C), or mixed, with prevalent membranous (M/C) or prevalently cytoplasmic (C/M) staining. For each case, the cumulative percentage of positive cells among all sections examined was determined. Interrate reliability between the two investigators blindly and independently examining the immunostained sections was assessed by the Cohen's *K* test, yielding *K* values higher than 0.70 in almost all instances.

### 2.3. Cell Lines

Cell lines derived from OSCCs and normal keratinocytes have been used for Real-Time PCR analysis. The cell lines used were (1) NCTC 2544 (normal and immortalized keratinocytes originated from skin keratinocytes of human origin and obtained from Interlab Cell Line Collection, Genova, Italy); (2) KB (CCL-17 dedifferentiated cell line, originated from human oral squamous cell carcinoma and obtained from ATCC, Manassas, VA, USA); (3) OSC-20 (well-differentiated oral SCC cell line, originated from a human oral squamous cell carcinoma and kindly supplied by Professor N. Tanaka and Dr H. Kondo, Sapporo Medical School, Sapporo, Japan); (4)-(5) CAL 33 and CAL 27 (moderately differentiated oral SCC cell lines, originated from a human squamous cell carcinoma and kindly supplied by Prof. J. L. Fischel, Centre Antoine-Lacassagne, Nice, France; these cells have relatively long doubling times (35 and 43 h, resp.)).

The cell lines were cultured as monolayers at 37°C in Dulbecco's Modified Eagles Medium (DMEM NCTC 2544, KB, CAL 27, and CAL 33) or RPMI 1640 (OSC20) supplemented with 10% fetal bovine serum (FBS), 0.075% sodium bicarbonate, and 0.6 mg/mL L-glutamine.

### 2.4. RNA Extraction

Total RNA was isolated by RNeasy minikit (Qiagen, Hilden, Germany), according to the manufacturer's instructions. The structural integrity of all tested total RNA samples was verified by agarose gel electrophoresis.

### 2.5. Reverse Transcription

Samples containing 5 *μ*g of total RNA in a final volume of 100 *μ*L were reversed-transcribed by avian myeloblastosis virus (AMV) reverse transcriptase (Promega, Madison, WI), according to the manufacturer's instructions. Random hexamer primers were used and the reaction was incubated for 60 min at 42°C. The ss-cDNA obtained was used for Real-Time PCR amplification.

### 2.6. Real-Time PCR

Real Time PCR has been used in order to perform quantitative analysis of beta-catenin in oral cancer cell lines, normal oral keratinocytes, and representative cases of oral cancer (22 cases with paired normal oral mucosa).

Real-Time PCR analysis of beta-catenin gene expression was performed by using the iCycler apparatus (BioRad, Hercules, CA) with sequence-specific primer pairs for the gene tested: respectively, beta-catenin *forward* GGGATGTTCACAACCGAATTGT and beta-catenin *reverse* GCTACTCTTTGGATGTTTTCAATGG. Amplification of the housekeeping gene glyceraldehyde-phosphate-dehydrogenase (GAPDH) from the same samples was used as internal control. The primers used were the following: GAPDH *forward*, 5′-TGG TAT CGT GGA AGG ACT CAT GAC-3′ and *reverse*, 5′-ATG CCA GTG AGC TTC CCG TTC AGC-3′. The cDNA was serially diluted and every dilution was run in triplicate. The Real-time PCR analysis was performed by using an initial denaturation step at 95°C for 3 min, followed by 50 cycles of denaturation at 95°C for 1 s, annealing at 50°C for 10 s, elongation at 72°C for 8 s. The IQ Sybr Green SuperMix (BioRad, Hercules, CA, USA) was used for Real-Time monitoring of amplification. Briefly, amplification was performed in a total volume of 20 *μ*L. The reaction mixture contained 10 *μ*L of 2x IQ Sybr Green SuperMix, 0.5 *μ*L of each primer (16 *μ*M), and 2 *μ*L of cDNA (or water as blank, which was always included). The Real-Time PCR products were electrophoresed on 2% agarose gel containing TAE (standard Tris-Acetate-EDTA electrophoretic buffer). The amplicons of expected size were extracted, purified, and controlled for sequence. Results were evaluated by means of ICYCLER IQ Real-Time Detection System Software (BioRad). Data were calculated on threshold cycle (Ct). We assumed ΔCt = beta-catenin Ct − GAPDH Ct, ΔΔCt = pathological ΔCt − normal tissue ΔCt, and the fold change of expression as 2_−ΔΔCt_. The last value represents the pathological versus normal mucosa gene expression. Differences in Ct values (ΔCt) reflect differences in copy number of mRNA molecules by the following formula: *N* copies × 2_Δcycles_. Mean values from three independent experiments were taken as results.

### 2.7. DNA Ploidy

Since we retain that the overall evaluation on DNA content should be performed only on histological cases rather than on cytological ones, our DNA ploidy analyses has been defined on 22 representative surgical samples (also analyzed by RT-PCR), in order to avoid the underestimation of the smallest cell fraction with aneuploid content. Four-*μ*m serial sections from each case of the 22 selected OSCCs, deparaffinized in xylene and rehydrated through decreasing concentrations of alcohols, were stained with Feulgen (sulfuric fuchsin-acid) for nuclear DNA staining. The quantitative DNA-image cytometric analysis was carried out by means of a visual computer analysis system (Quantimet 500 IW analyzer, Leica, Cambridge, England; 3CCD camera DXC-95OP, Sony, Köln, Germany; Leica QWINVO200A and Leica Qploidy software). At least 200 cancer cells for each case were examined in nonconsecutive random fields of representative areas of the lesions. By using a minimum of 30 lymphocytes the following parameters were determined: (1) the normal diploid DNA content (2c); (2) the DNA deviation index (DI); (3) the 2c DI, defined as the average quadratic deviation of the diploid value; (4) the percentage of cancer cells with a DNA content higher than 5c; (5) the DNA malignancy grade. Lesions were classified as follows: (1) diploid, when more than 70% of cells with a DI ranging from 0.8 to 1.12 were detected; (2) tetraploid, when 20% or more of cells with a DI ranging from 0.8 to 1.12 and 5% or more of cells in the range 2.12 to 4.12 were found; (3) aneuploid, when 10% or more of cells with a DI distributed in all other values outside the range 1.12 to 1.8 and/or 20% or more of cells with a DI ranging from 1.12 to 1.8 were observed. The different degree of aneuploidy has been identified according to Duesberg P. classification: *near diploid* class with low instability, 1.5 *N* class with high instability, *near* 3 *N* class with very high instability [31].

### 2.8. Statistical Analyses

The data were analyzed by the Stanton Glantz statistical software 3 (MS-DOS) and Graph-Pad Prism software version 4.00 for Windows (Graph Pad software San Diego California, USA; http://www.graphpad.com/). Differences between groups were determined using the one-way analysis of variance (ANOVA) and the Student-Newman-Keuls test to evaluate the correlation between membranous, nuclear, and cytoplasmic expression of beta-catenin and clinical-pathological parameters. *P* values <0.05 were regarded as significant.

Therefore, ordinal logistic regression was performed by SPSS 10.1 software, in order to verify the association between the presence and the impact of the two major risk factor (tobacco and alcohol, as covariates) and the different topographical intracellular pattern of beta-catenin expression (response variable) [32]. Kaplan-Meier curves were plotted for the survival analysis of the patients. 

## 3. Results and Discussion 

### 3.1. Study Population

To our knowledge, the present study is the largest retrospective analysis that investigates the association between expression of beta-catenin and the traditional clinicopathological parameters in oral and oropharyngeal carcinomas. Patients from distinct geographical areas and different ethnic groups (Caucasians, Blacks, and Hispanics) were included. Demographical and clinicopathological characteristics, summarized in Table 1, revealed a large population of 374 cases of squamous cell cancers, distinct for stage, histological grade, and sites. The TNM tumour extent of all cases as determined based on the clinic-pathological information (X-ray, bone scan, computed tomography, and pathological reports) was as follows: 86 cases in stage I, 72 in stage II, 124 in stage III and 92 in stage IV. Based on the histopathological examination, 62 tumors were G1, 188 were G2, and 124 were G3. Furthermore, 233 patients underwent surgery; 59 patients received surgery and radio-/chemotherapy only with curative intention; 82 patients have been treated with radiotherapy alone. During the follow-up period (with a mean of 39.41 months), 142 patients remained tumour-free and 207 patients died from disease, whereas, in 25 cases, death was from other causes independent of their cancer.

### 3.2. One-Way ANOVA Analysis

**Figure 1:**
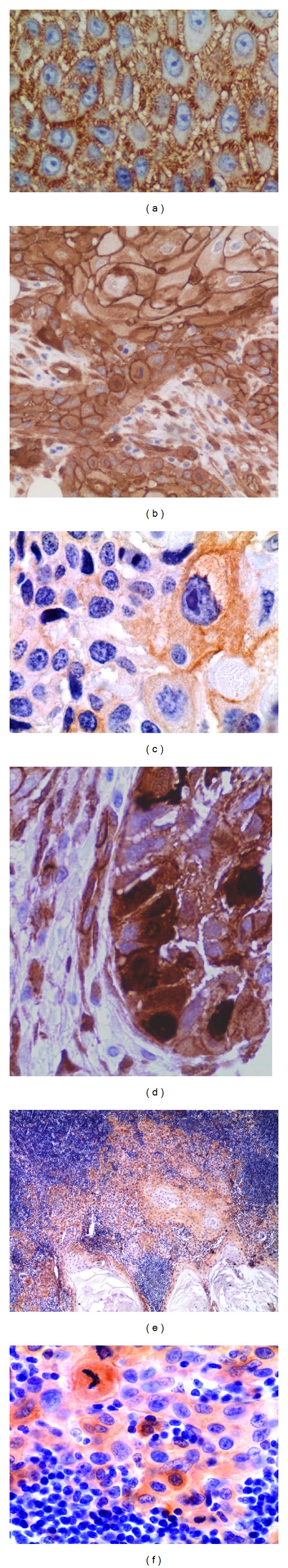
Immunohistochemical expression of beta catenin in oral cancer. (a) Normal membranous staining in oral epithelium surrounding OSCC (x400); (b) strong cytoplasmic increase with membranous overexpression in OSCC (x200); (c) cytoplasmic downregulation expression (x400); (d) strong nuclear cytoplasmic expression (x200); (e) lymph node metastasis from OSCC showing cytoplasmic expression (x40); (f) further detail of cytoplasmic expression in lymph node metastasis (x200); LSAB-HRP technique, nuclear counterstaining with haematoxylin.

Immunohistochemical examination revealed a significant difference in beta-catenin expression between cancerous cells and normal epithelium (*P* < 0.05). More precisely, normal mucosa surrounding tumors displayed a characteristic membranous pattern of protein expression whereas OSCC/OPSCC cancerous cells exhibited cytoplasmic and focal nuclear beta-catenin localization (Figure 1). Only one case, described as basaloid variant of squamous cancer, has shown a marked and diffuse nuclear staining.

Our findings have revealed the following various immunohistochemical patterns.

**Figure 2:**
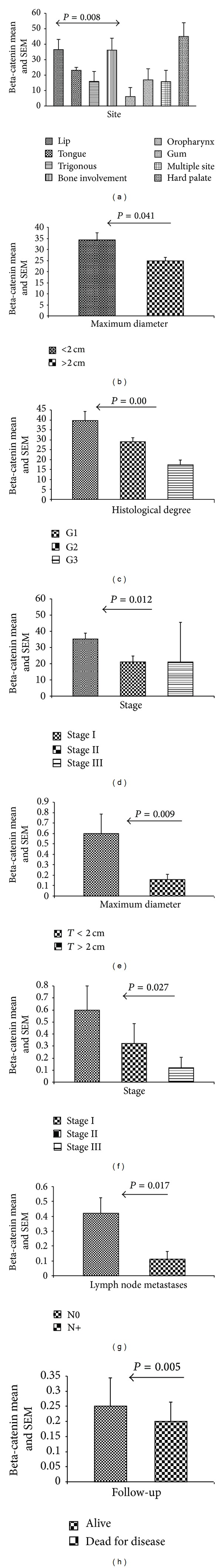
Persistent detection of membranous staining for beta-catenin (a)–(d) and sporadic nuclear staining for beta-catenin (e)–(h). Membranous expression of the study marker was more observable in the lip and palate carcinomas (M, *P* = 0.008) (a), in <2 cm tumor (*P* = 0.041) (b), and in well-differentiated cancers (*P* = 0.00) (c), in early stage (St I) tumours (*P* = 0.012) (d). Sporadic nuclear positivity for beta-catenin was more observable in <2 cm tumor (*P* = 0.009) (e), in early stage (St I) tumours (*P* = 0.027) (f), without nodal metastases (N0) (*P* = 0.017) (g), and with a better clinical followup (*P* = 0.005) (h).

**Figure 3:**
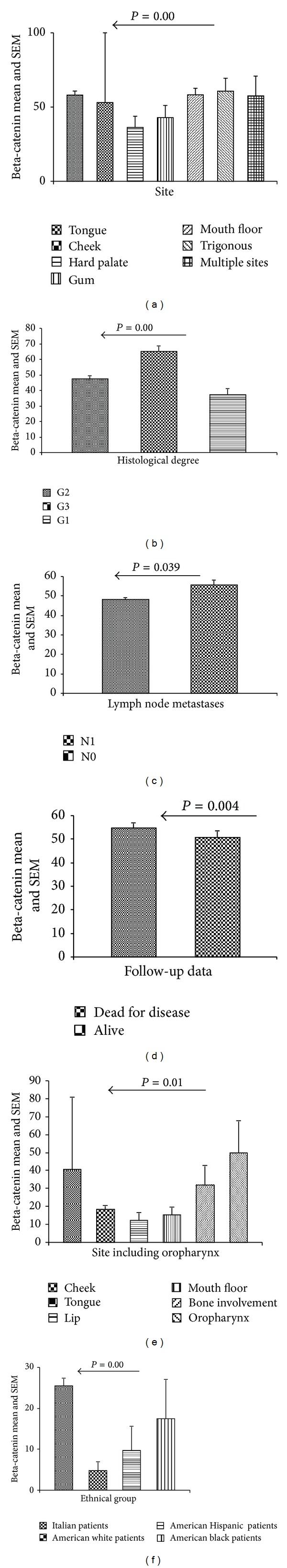
Cytoplasmic delocalization with high expression of beta-catenin (a)–(d) and cytoplasmic delocalization of beta-catenin with immunostaining absence of marker (e)-(f). Cytoplasmic delocalization with high expression of beta-catenin has been prevalently observed in OSCCs/OPSCCS of the tongue, trigonous, gingival mucosa, jaw, and in the tumors sited on the floor of the mouth, on the alveolar ridge, and, finally, in the multifocal neoplasias (*P* = 0.00) (a). This immunohistochemical pattern was also observed in undifferentiated carcinomas (*P* = 0.00) (b), with clinical history of lymph nodal metastases (N+) (*P* = 0.039) (c), and with worse prognosis (*P* = 0.004) (d). Cytoplasmic delocalization with loss of beta-catenin was, generally, reported in oropharyngeal carcinomas and cheek cancer (*P* = 0.01) (e), in Italian patients (*P* = 0.00) (f).

- *Persistent detection of membranous staining for beta-catenin* was more common in the lip and palate-originated carcinomas (M, *P* = 0.008) (Figure 2(a)), in <2 cm tumor (*P* = 0.041) (Figure 2(b)), in well-differentiated cancers (*P* = 0.00) (Figure 2(c)), in early stage (Stage I) tumors (*P* = 0.012) (Figure 2(d)), and in patients nonusers of alcohol and/or tobacco (*P* = 0.045).
- *Sporadic nuclear positivity for beta-catenin* was predominantly observed in <2 cm tumor (*P* = 0.009) (Figure 2(e)), in early stage (Stage I) tumors (*P* = 0.027) (Figure 2(f)), without nodal metastases (N0) (*P* = 0.017) (Figure 2(g)), and in patients nonusers of alcohol and/or tobacco (*P* = 0.045), with a better clinical outcome (*P* = 0.005) (Figure 2(h)).
- *Cytoplasmic delocalization with high expression of beta-catenin* has been prevalently observed in OSCCs/OPSCCS of the tongue, trigonous, gingival mucosa, jaw, floor of the mouth, alveolar ridge, and, finally, in multifocal cancers (*P* = 0.00) (Figure 3(a)). This immunohistochemical pattern was also observed in undifferentiated carcinomas (*P* = 0.00) (Figure 3(b)), with lymphnode metastases (N+) (*P* = 0.039) (Figure 3(c)), and with the worse clinical prognosis (*P* = 0.004) (Figure 3(d)).
- *Cytoplasmic delocalization with loss of beta-catenin* was generally observed in oropharyngeal and cheek carcinomas (*P* = 0.01) (Figure 3(e)), in Italian patients (*P* = 0.00) (Figure 3(f)), with clinical history of tobacco, and/or alcohol consumption (for the two risk factors combined, *P* = 0.00; for the tobacco use only, *P* = 0.023; for the alcohol consumption only, *P* = 0.00).

### 3.3. Multiple-Way ANOVA

Comparing dependent variable beta-catenin expression to different independent variables as gender, ethnic group, age, anatomic sites, tumor diameter, histological grade, T, N, M, type of treatment, and presence of local relapse, we have also performed a multiple-way ANOVA that revealed the following findings that were statistically significant. Various levels of membranous and cytoplasmic beta-catenin expression were observed according to the ethnicity, anatomic site, histologic grade, and type of treatment. Absence of staining was found in great percentage in Caucasians (25.49%). Various levels of beta-catenin nuclear expression were observed according to tumor size and presence of local recurrence.

### 3.4. Ordinal Logistic Regression

Ordinal logistic regression confirms that tobacco and alcohol-related cancers are associated to delocalization with complete loss of expression of the studied marker. In contrast, the nuclear, cytoplasmic and membranous beta-catenin expression seems not to be correlated with the exposure to these risk factors in oral and oropharyngeal carcinogenesis.

### 3.5. Survival Analyses

**Figure 4:**
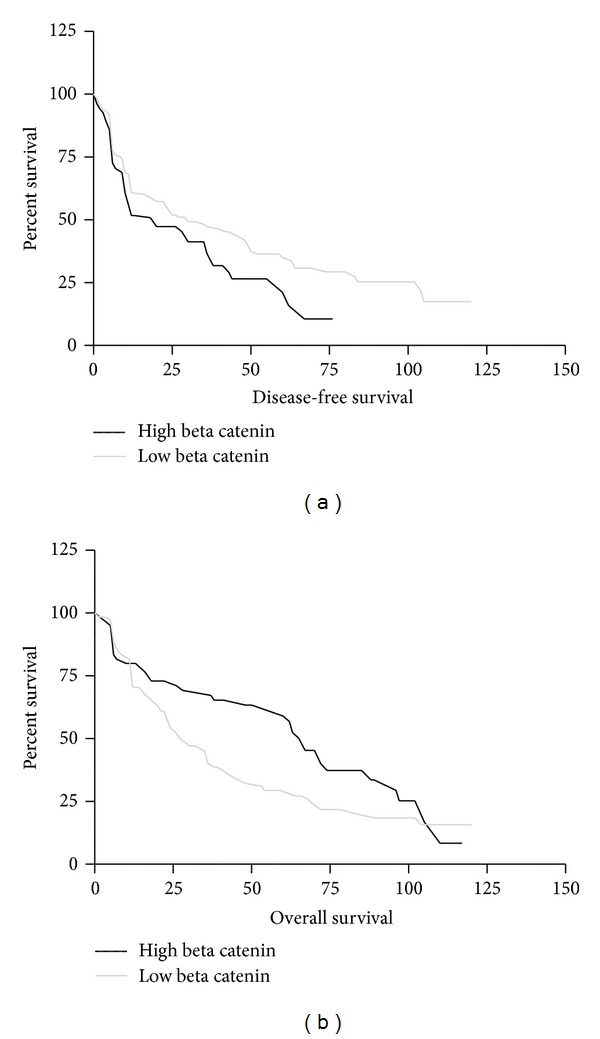
Kaplan-Meier curves ((a) overall survival and beta-catenin expression; (b) disease free survival and beta-catenin expression). Survival estimates have shown that OSCCS/OPSCCs overexpressing beta-catenin (cut-off >60%) are characterized by a worse prognosis, in terms of overall survival (*P* < 0.0153; survival median of 28 months in high expressing tumors versus 66 months in low expressing OSCCs/OPSCCs) (a) and disease-free survival, with a more evident tendency to relapse and metastasis (*P* < 0.0254; survival median of 20 months in high expressing tumors versus 30 months in low expressing OSCCs/OPSCCs) (b).

Based on the survival analyses and as confirmed by the Kaplan Meyer curves, our study has revealed the tumoral stage as a classic clinicopathologic parameter with significant prognostic value. More precisely, patients with advanced stages carcinomas had a higher mortality than patients with localized neoplasms (data not shown). Overall survival curves have also revealed that primary tumours of the trigone, floor of the mouth, tongue, and multiple sites expressed the higher levels of morbidity (data not shown). By Kaplan-Meier curves, survival estimates have shown that OSCCS/OPSCCs overexpressing beta-catenin (cut off >60%) are characterized by a worse prognosis, in terms of overall survival (*P* < 0.0153; survival median of 28 months in high expressing tumors versus 66 months in low expressing OSCCs/OPSCCs) (Figure 4(a)) and disease-free survival, with a more evident tendency to relapse and metastasis (*P* < 0.0254; survival median of 20 months in high expressing tumors versus 30 months in low expressing OSCCs/OPSCCs) (Figure 4(b)). Moreover, the Kaplan-Meier curves have also underlined the statistically significant association between beta-catenin overexpression, high degree of differentiation (OS, *P* < 0.0002; DFS, *P* < 0.0004), and advanced stage (OS and DFS, *P* < 0.0001) (data not shown).

### 3.6. RT-PCR (Quantitative) on Cell Lines

**Figure 5:**
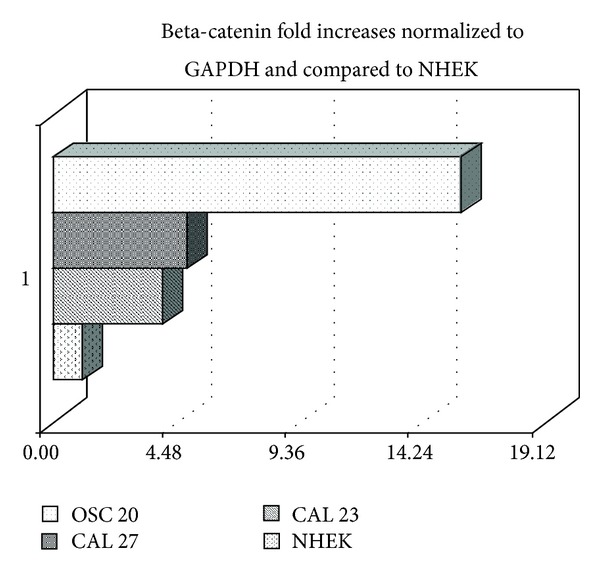
Beta catenin overexpression in OSCC cell lines as evaluated by Real Time PCR. The Δ value was calculated by determining the mean value of beta-catenin expression by Real Time PCR. Beta-catenin showed upregulation in OSC20 cell line derived from aggressive cancer compared to normal epithelial cell line.

The Δ value was calculated by determining the mean value of beta-catenin expression by Real Time PCR. Beta-catenin expression showed upregulation in OSC20 cell line derived from aggressive cancer compared to normal oral epithelial cells (Table 2) (Figure 5).

### 3.7. RT-PCR (Quantitative) on Surgical Samples

By Real Time PCR, variations of beta-catenin mRNA expression in OSCCs/OPSCCs are present in 72% of the examined cases. In 10 out of 22 cases (45%) a significant increase of beta-catenin mRNA was observed. Interestingly, among these 10 overexpressing beta-catenin cases, 50% had lymphnode metastases. On the other hand, 27% of tumors displayed a decrease in beta-catenin mRNA expression.

### 3.8. DNA Ploidy

**Figure 6:**
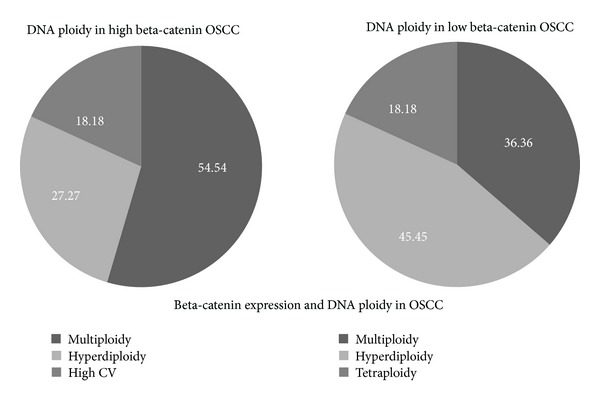
Beta-catenin and DNA ploidy in OSCC. Two OSCCs groups were obtained by RT-PCR esteeming: (i) high-expressing beta-catenin cancers (a) showing high aneuploidy degree (prevalently multiploid, followed by hyperdiploid and highly heterogeneous tumors) and lymph node metastases in T1 tumors; (ii) low-expressing beta-catenin cancers (b) with lower aneuploidy degree (prevalently hyperdiploid, followed by lower proportion of multiploid tumor, and tetraploid ones) and no lymph node metastases in T1 tumors.

In our analysis, almost all the cases displayed different grade of aneuploidy, from hyperdiploidy to tetraploidy, multiploidy, and hypodiploidy. Euploid cancers represent only a minority (9.67%). Therefore, our study on DNA *ploidy* has revealed a potential association between beta-catenin *upregulation* and the altered DNA content in cancer samples. Two OSCCs groups were identified by the RT-PCR evaluation (Figure 6):

- *high-expressing beta-catenin cancers* showing high aneuploidy degree (prevalently multiploid, followed by hyperdiploid and highly heterogeneous tumors) and lymph node metastases in T1 tumors,
- *low-expressing beta-catenin cancers* with lower aneuploidy degree (prevalently hyperdiploid, followed by lower proportion of multiploid and tetraploid tumors) and no lymph node metastases in T1 tumors.

Squamous cell carcinoma is the most frequent malignant tumor of the oral and oropharyngeal cavity [26, 33]. The five-year survival rate for patients with OSCC/OPSCC in developed countries still remains very poor (of a dismal 40–50%) and has not improved over the past decades, despite advances in detection and therapy. Thus preventive approaches before the development of invasive cancer are highly desirable and novel strategies to reduce cancer incidence are currently pursued worldwide. The molecular mechanisms involved in the OSCC/OPSCC carcinogenesis are not well understood, nor their genetic profile has been fully characterized [34–36]. Abnormal cell adhesion is a peculiar feature of cancer diseases and represents a crucial point in tumor development and progression. Beta-catenin, taking part in the tightly orchestrated WNT pathway, is a crucial protein able to coordinate the expression and function of several downstream molecules [37]. It is well known that alteration in beta-catenin expression may also lead to aneuploidy and chromosome aberrations [31, 38–40]. Beta-catenin is widely expressed in proliferating normal epithelia and in numerous human cancers [7]. Recent studies have identified beta-catenin as a cancer-susceptibility gene [41]. Any type of aberration at different levels (genic mutation, epigenetic alteration of WNT pathway members) may lead to protein overexpression, detectable in the nucleus of numerous malignant cells, thus resulting in abnormal activation of cell proliferation and migration [42, 43].

In a recent work [27] we demonstrated that other mechanisms, different from genic mutation, such as epigenetic methylation of WNT inhibitors, can induce the constitutive activation of WNT signal with intracellular accumulation of beta-catenin. Moreover, various metabolic pathways, besides the well-known WNT, can determine the intracellular delocalization of beta-catenin. In fact, various studies have reported that EGF and TGF*β* can induce the phosphorylated form of p68 to bind beta-catenin, resulting in nuclear translocation of the latter with the activation of numerous downstream genes. According to other studies, MET, Fer, or Fyn kinase can also cause beta-catenin phosphorylation on Tyr 142 residue and its nuclear translocation. In fact, in this activated status beta-catenin would lose bond affinity for E-cadherin and alpha-catenin, increasing the strength of association with the transcriptional factor BCL9-2 [21]. Wilding et al. [44] showed that EGFR overexpression correlates with perturbation of the E-cadherin/catenin complex seen in the human papillomavirus HPV-16 E6- and E7-transfected keratinocytes, which may indicate a possible functional interaction between growth regulatory factors and adhesion molecules, particularly in a subset of virus infected cancers.

Considering all these data, we can postulate that beta-catenin could be involved in different molecular pathways and that other proteins could act as key components in the activation of the Wnt/beta-catenin signaling that occurs during OSCC/OPSCC cancer progression. Reduced levels of membrane-bound E-cadherin and/or *γ*-catenin and cytoplasmic accumulation of beta-catenin have been often observed in OSCCs/OPSCCs, indicating that Wnt signaling is activated in this disease and suggesting that the activation could be via an alternative pathway [30, 45].

Several groups have reported that the intracellular distribution of beta-catenin is different in oral neoplastic cells than in normal mucosa. Whereas beta-catenin is localized exclusively to the cell membrane in normal oral mucosal epithelium, this membrane-bound expression is absent or diminished in neoplastic cells, associated with a corresponding increase in its cytoplasmic localization [23, 24, 26].

Our work has highlighted the presence of four different immunohistochemical patterns: (1) persistent detection of membranous staining for beta-catenin, predominantly in G1 cancers, in stage I tumors, and in patients unexposed to alcohol and tobacco; (2) sporadic nuclear positivity for beta-catenin in early stage (St I) tumors and in patients unexposed to alcohol and tobacco, with a better clinical followup; (3) cytoplasmic delocalization with high expression of beta-catenin, observed in undifferentiated carcinomas with clinical positivity of lymph node metastases and with worse prognosis; (4) cytoplasmic delocalization with loss of beta-catenin, predominantly in oropharyngeal carcinomas with clinical history of tobacco and/or alcohol consumption. Ordinal logistic regression confirmed that tobacco and alcohol-related cancers are associated to beta-catenin complete loss of expression. Nuclear, cytoplasmic, and membranous expression of beta-catenin seems not to be associated with tobacco and/or alcohol consumptions, well-known risk factors in oral and oropharyngeal carcinogenesis. Therefore, we postulate that, in these patterns of protein expression, other carcinogenetic events may play a role in the intracellular distribution of beta-catenin.

There is substantial epidemiologic and molecular evidence to support the causal association between human papillomavirus (HPV) and a subset of oropharyngeal cancers [46–48]. Activation of Wnt pathway by viral oncoproteins has been already reported for Epstein Barr virus (EBV) and Kaposi's sarcoma-associated herpesvirus [49]. In the case of Kaposi's sarcoma-associated herpesvirus, it is the encoded latency-associated nuclear antigen protein that regulates the nuclear accumulation of GSK3-*β* [50]. In EBV-associated nasopharyngeal carcinoma, nuclear accumulation of beta-catenin has been proposed to occur through the activation of the phosphoinositide 3-kinase/AKT pathway by latent membrane proteins (LMP) 1 and 2A EBV proteins [51]. In a recent work Rampias et al. detected increased beta-catenin transcriptionally active protein levels in HPV16-positive-associated oropharyngeal squamous cell [52]. They also observed that, in HPV16-negative oropharyngeal squamous carcinomas, beta-catenin was mainly localized at the cell membrane. On the other hand, in HPV16-positive oropharyngeal squamous carcinomas, beta-catenin was detected in the cytoplasm and nucleus. In the present study we have considered as risk factors for OSCC/OPSCC only the presence/absence of tobacco/alcohol consumption and by ordinal logistic regression we have correlated them with a specific pattern of protein expression. In previous works, we have also investigated the prevalence on HPV virus in oral and oropharyngeal cancers [53], the golden standard method of HPV detection [54], and the prognostic role of other important proteins that are functionally interacting with beta-catenin [55–57]. The complex molecular mechanism by which beta-catenin relates to other parameters with potential prognostic or diagnostic value like viruses (e.g., HPV) and various oncogenes and tumor suppressor genes (p16, EGFR, p53, etc.) should make the object of further investigations on a large study sample.

The current literature on the prognostic role of beta-catenin has been limited by small series with either short follow-up period or lacking detailed clinic-pathologic data. This current study confirms and extends our previous findings that have indicated that beta-catenin displayed intracellular delocalization in OSCCs, which was correlated with development of local recurrences [30].

To our knowledge, the present study is the largest retrospective analysis that investigates the association between expression of beta-catenin and the traditional clinicopathological parameters in OSCC/OPSCCs of different grade and stage. This study combines different molecular diagnostic methods, such as immunohistochemistry, Real-Time quantitative RT-PCR, and DNA cytometric analysis, in tumor specimens and cell lines. Using this combinatory approach, we have revealed a prognostic value for cytoplasmic and nuclear beta-catenin. Our results obtained from quantitative RT-PCR and DNA cytometric analyses let us postulate that the abnormal beta-catenin intracellular delocalization could be associated with a higher aneuploidy degree, in support of its known role in chromosomal instability.

Cancer of the oral cavity and oropharynx remains a complex problem in pathogenesis, prevention, and treatment and there is a critical need for accurate prognostic biomarkers. Our line of investigations provides strong evidences in support of the prognostic value of beta-catenin and its relevance to the clinical practice in terms of diagnostic strategy and management of OSCCs/OPSCCs to early differentiate among various pathological entities. Further studies on independent series are required in order to establish a significant prognostic role for beta-catenin expression in these types of cancers.

## 4. Conclusions

Our study focuses on better understanding the crucial role of beta-catenin in OSCC/OPSCC carcinogenesis. Our findings suggest that intracellular translocation of beta-catenin may be important in the activation of Wnt signaling in OSCC/OPSCC cancer, resulting in enhanced tumor growth and possibly more aggressive tumor behavior. There is increasing evidence that elevated beta-catenin expression in neoplastic cells may also contribute to their cancer stemness potential [58, 59]. In OSCCs/OPSCCs, delocalized and overexpressed beta-catenin might identify a small cell fraction with proliferative, maybe staminal, neoplastic characteristics and a tumoral group with aggressive behavior and poor prognosis. Thus, intracellular accumulation of beta-catenin expression may be an important predictor of clinical outcome for patients with OSCC/OPSCC cancer. These results could help towards development of better diagnostic and targeted therapeutic approaches that will ultimately lead to better patient survival with more personalized and less toxic treatment.

## Figures and Tables

**Table 1:** Clinicopathological characteristic of study population.

Age					
Range			33–98		
Mean			65,34		
Sex					
Male			289		
Female			85		
Ethnic group					
Caucasian White			286		
American White			61		
Hispanic			19		
American Black			8		
Site					
Trigonous (Tr)			14		
Tongue (T)			168		
Gums (G)			18		
Cheek (C)*			34		
Lip (L)			29		
Bone Involvement (BI)**			23		
Oropharynx (OP)			4		
Palate (P)			12		
Floor of the mouth (FOM)			46		
Multiple sites (MS)***			11		
Metastasis (Met)****			15		
Total			**374**		
Maximum diameter (cm)					
<2 cm			102		
>2 cm			272		

TNM staging					

T	N0 M0	N0 M1	N1 M0	N1 M1	Total

T1	86	3	25	3	**117 **
T2	72	0	57	7	**136**
T3	13	0	29	8	**50 **
T4	25	1	39	6	**71**
Total	**196**	**4**	**150**	**24**	**374**

Stage					
St1			86		
St2			72		
St3			124		
St4			92		
Histologic grade					
G1			62		
G2			188		
G3			124		
Follow-up					
Alive			142		
Dead for disease			207		
Dead for other causes			25		
Local recurrence					
No			215		
Yes			159		
Therapy					
CH			233		
CH-RT/CH-RT-CHT			59		
RT			82		

Legend: *Cheek includes gingival mucosa.

**Bone involvement of mandible or upper alveolar process.

***Multiple sites includes FM + Tr, T + Tr, T + G, C + L, T + C, T + L.

****Metastases analyzed without primary tumors due to not operable advanced stages primary cancer.

**Table 2:** Significant fold increase of beta-catenin in cancer derived cell lines as evaluated by Real Time RT-PCR.

Cell lines	ΔCt beta-catenin	2^Δ−ΔDeltaCt^
(1) Cal 27	2.36	5.13
(2) Cal 33	2.06	4.16
(3) KB	2.16	4.45
(4) OSC20	3.96	15.56

(5) NCTC	5.36	0.25
